# Fault Tolerant Coverage and Connectivity in Presence of Channel Randomness

**DOI:** 10.1155/2014/818135

**Published:** 2014-01-21

**Authors:** Anil Kumar Sagar, D. K. Lobiyal

**Affiliations:** School of Computer and Systems Sciences, Jawaharlal Nehru University, New Delhi 110067, India

## Abstract

Some applications of wireless sensor network require *K*-coverage and *K*-connectivity to ensure the system to be fault tolerance and to make it more reliable. Therefore, it makes coverage and connectivity an important issue in wireless sensor networks. In this paper, we proposed *K*-coverage and *K*-connectivity models for wireless sensor networks. In both models, nodes are distributed according to Poisson distribution in the sensor field. To make the proposed model more realistic we used log-normal shadowing path loss model to capture the radio irregularities and studied its impact on *K*-coverage and *K*-connectivity. The value of *K* can be different for different types of applications. Further, we also analyzed the problem of node failure for *K*-coverage model. In the simulation section, results clearly show that coverage and connectivity of wireless sensor network depend on the node density, shadowing parameters like the path loss exponent, and standard deviation.

## 1. Introduction

Wireless sensor network (WSN) consist of low-cost small sensor nodes equipped with small sensing, communication, and computation capabilities. These nodes are deployed either deterministically or stochastically and sense the events of interest and send this data to one or more sink. There are various applications of WSN which includes battlefield surveillance, environmental monitoring, health care, and vehicle traffic monitoring [1]. In hostile environments, where human intervention is difficult or impossible, sensors may be dropped randomly from an airplane. In this case, node density cannot be the same in the whole area. If less number of sensors are deployed, some area may be uncovered and some nodes may be isolated. To maintain quality of coverage and connectivity, large number of sensors are deployed. These redundant sensors improve coverage as well as connectivity but increase energy consumption and network cost which eventually reduces network lifetime. Quality of monitoring of a given area is a fundamental issue in many applications, which measures how well a sensor monitors the given target area. Therefore, coverage is one of the key factors to achieve quality of service in a wireless sensor network. Covering each point by only one sensor is desired in some applications, while in many applications, it is desired that more than one sensor can cover each point in the target area for better accuracy and fault tolerance. To deal with the problem of faulty sensors, some applications required *K*-coverage; that is, every location in the field is covered by at least *K* sensors. Connectivity is another factor that affects the quality of service. It enables the sensors to communicate with each other so that their sensed data can reach the sink. To ensure data delivery, multiple paths between a source and destination may be available. Therefore, if there are *k* disjoint paths exist between two sensors, such a network is called *K*-connected. In other words, a network is *K*-connected, if any *K*-1 nodes are randomly chosen and removed from the network, and it still remains fully connected. Therefore, in this paper, we are concerned with the *K*-Coverage and *K*-Connectivity problem which requires that every point in the bounded field is monitored by at least *K* sensors at any point of time and there exists *K* independent paths from a source to destination. In previous work, the sensing and transmission range of sensor nodes is assumed to be based on disk model; that is, it is assumed to be symmetric in all direction which is not correct in real environments. Generally, it is viewed that sensing and transmission range of the sensor nodes is affected by distance and obstacles present in the environments; this phenomenon is known as shadowing effect. This paper also investigates the impact of shadowing model on *K*-Coverage and *K*-Connectivity of wireless multihop networks where each point in the target region is covered by at least *K* sensors and there are at least *K* paths from source to destination. The rest of the paper is organized as follows. The earlier work related to coverage and connectivity done by various researchers is given in Section 2. The proposed system model, sensing model, and log-normal shadowing model are presented in Section 3. Further, in Section 4,  *K*-coverage sensing affected by lognormal shadowing model is described.  Section 5 explains *K*-connectivity along with the effect of shadowing model. In Section 6, the effect of node failure on coverage is discussed. Simulation results and performance analysis of the model are presented in Section 7. The work in the paper is concluded in Section 8.

## 2. Related Work

Coverage and connectivity jointly had drawn the attention of researchers working in the design of multihop WSN. Several extensive studies related to coverage and connectivity in WSN have been proposed in the literature. However, most of the works have their basis on binary disk model of the channel propagation where the irregularity and randomness present in radio communications due to various environment effects are not considered.

Ammari and Das in [2] analyzed the problem of *K*-coverage. In this paper, authors studied sensors duty cycling strategies so that in each scheduling round each and every location of the sensor field is monitored by at least *K* active sensors and all these active sensors are also connected. Authors have also shown the relation between sensing and communication range for *K*-coverage and connectivity among all active sensors. In [3], Cai et al. proposed a sleeping protocol named area-based collaborative sleeping (ACOS) which saves energy as well as maximizes the coverage. In ACOS, a sensor can be either in passive, active, preactive, or prepassive state and each sensor can calculate its surface area which is not covered by any other sensor. Esnaashari and Meybodi in [4] suggested automata-based deployment strategy, called extended cellular learning automata-based deployment strategy (CLA-EDS), for *K*-coverage with mobile sensor nodes. This approach also calculates different degree of coverage in different region of the network. CLA-EDS deployment strategy does not use any information regarding the position of sensor nodes. In this approach, each node finds out its best position in cooperation with its neighboring nodes to attain high coverage. A partitioned synchronous network (p-sync) for coverage and connectivity together is studied by He et al. in [5]. To conserve energy, they used duty cycled approach in which sensor nodes are partitioned into number of disjoint subset. Authors also find out the optimal schedule for p-sync network and showed that connectivity is better in p-sync network rather than in synchronous network. Li et al. in [6] studied the problem of *k*-connected target coverage which is fault tolerant, by using minimum number of active nodes. They have used two heuristics algorithms (TS) and (RA). TS algorithm covers all the targets using set cover algorithm and adds some new nodes to form *k*-connected coverage. In RA algorithm, a sensor node goes in sleeping mode if there is no effect on coverage and any two neighbors of the node have *k* node-disjoint paths. Bai et al. in [7] discussed the multiple coverage with optimal locations of sensor nodes. They also proved that the optimal congruent deployment density for 2-coverage is $4\pi /3\sqrt{3}$. Dhillon and Chakrabarty in [8] proposed an optimization problem on sensor placement. They proposed a greedy heuristic to provide sufficient coverage with minimum number of sensor nodes. They have also proposed polynomial-time algorithms for placement of minimum number of sensor nodes. In [9], authors studied the impact of under log-normal shadowing model on connectivity where sensor nodes are scattered according to homogeneous Poisson process over an infinite plane. The authors also provided tight lower bounds for minimum node density so that the network is surely connected. They further analyzed how fading affected the topology of multihop networks. Finally authors validated their analytical result with simulation. Hekmant and Van Mieghem [10] have analyzed the link probability in wireless ad hoc networks using geometric random graph model. To study the connectivity, authors used more realistic log-normal shadowing models.

## 3. System Model

In the system model, we have considered that *N* sensors are deployed randomly and uniformly in the two-dimensional desired sensing area. These sensor nodes are static and homogeneous and deployed with high density according to a homogeneous Poisson distribution with density *λ*. Each sensor with radius *r*_max_ can cover a circular area *πr*_max_^2^ while ignoring the border effect. The area of interest is said to be covered by sensors if every point of interest lies within the sensing range of at least one sensor node. Each sensor node detects the event of interest and reports this to the sink. It is assumed that all nodes are equipped with transmitter and receiver having the same features; thus, we have a single value of sensing range for all nodes. However, the sensing range of each sensor nodes depends on fixed transmission power *P*_*s*_, and propagation loss due to distance and environment. All sensors are aware of their locations using some localization technique [11]. System parameters and their definitions are listed in Table 1.

### 3.1. Sensing Model

Sensing coverage generally uses the binary disk model where each sensor's coverage area is modeled by a disk. It is assumed that sensing area of a sensor is circle and sensor nodes sense in all direction uniformly; that is, it is omnidirectional. A sensor node detects an event of interest with probability one if it is in the sensing range of the sensor node and with probability zero otherwise. The target at point *p* can be detected by the sensor *s* if and only if *d*(*s*, *p*) ≤ *r*_max_. Depending on whether the event is located within the sensing range, the coverage function of the binary disk model is formulated as the sensing function *f*(*d*(*s*, *p*)) given below

(1) $$\begin{array}{c} f\left(d\left(s,p\right)\right)=\{\begin{array}{cc} 1, & \text{if}\;d\left(s,p\right)\leq r_{\max},\\ 0, & \text{if}\;d\left(s,p\right)>r_{\max}. \end{array} \end{array}$$

**Figure 1:**
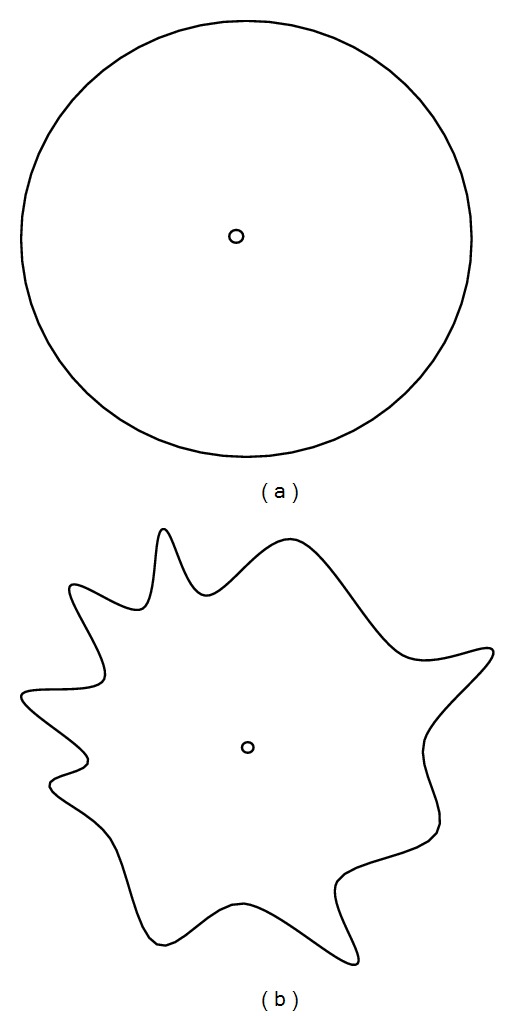
(a) Transmission range in ideal case. (b) Transmission range with impact of path loss and shadowing.

In the function, *d*(*s*, *p*) is the Euclidean distance between sensor node *s* and a point *p*; and *r*_max_ is sensing range of a sensor node.  Figure 1(a) shows the transmission range of binary disk model which is assumed to be isotropic. Probabilistic sensing model is much more realistic than binary sensing model. The probability of event detection by a sensor node is dependent on the sensor-event distance. As the distance between sensor and event increases, the strength of sensing signal may drop along the path. In the probabilistic sensing model, the sensing function *f*(*d*(*s*, *p*)) can be modeled as

(2) $$\begin{array}{c} f\left(d\left(s,p\right)\right)=\{\begin{array}{cc} 1 & \text{if}\;d\left(s,p\right)\leq r_{u}\\ e^{-\alpha d{\left(s,p\right)}^{\beta }} & \text{if}\;r_{u}<d\left(s,p\right)\leq r_{\max}\\ 0 & \text{otherwise}, \end{array} \end{array}$$

where *α* and *β* are sensor-dependent parameters which represents the physical characteristic of the sensor, *r*_*u*_ is the starting of uncertainty in sensor detection, and *r*_max_ is the maximum practicable sensing range of sensor node. In real networks, radio fluctuations, obstacles, and noise cannot be ignored causing the random signal strength; this effect is called shadowing. Random attenuation due to shadowing is modeled as log-normal. Thus, log-normal shadowing is the most widely accepted model that considers shadowing effect. This model shows that average received signal strength decreases logarithmically with respect to distance between transmitter and receiver. Therefore, due to distance and shadowing effect, the sensing ability of sensors is not the same in all the directions (Figure 1(b)).

### 3.2. Log-Normal Shadowing Path Loss Model

This model represents radio propagation in real environment. Path loss is caused by dissipation of the power radiated by the transmitter as well as by the effects of fading and shadowing that is caused by impediments present between the transmitter and receiver. According to this model, both theoretical and measurement-based propagation models indicate that the average received signal power decreases logarithmically with the distance [12]. In this radio propagation path loss model, path loss PL(*d*) corresponding to distance *d* is described as

(3) $$\begin{array}{c} \text{PL}\left(d\right)=\text{PL}\left(d_{0}\right)+10\eta \log\left(\frac{d}{d_{0}}\right)+X_{\sigma }, \end{array}$$

where PL(*d*_0_) is path loss corresponding to reference distance *d*_0_, *η* is the path loss exponent that shows the rate of increasing path loss with distance, and *X*_*σ*_ expresses log-normal shadowing effect which is the Gaussian random variable with zero mean and standard deviation *σ*. The values of *η* and *σ* are computed from measured data. This model assumes that path loss has same value when measured in different direction. However, Zhou et al. [13] showed experimentally that path losses should be nonisotropic due to many factors such as sending power, antenna gain, receiver sensitivity, signal-to-noise ratio, and obstacle present in the environment. Therefore, based on experiment of [13], path loss expressed in (3) will be

(4) DOI−adjusted  path  loss  (PL(d)DOI)  =path  loss(PL(d))×ki,

where

(5) $$\begin{array}{c} k_{i}=\{\begin{array}{cc} 1 & \text{if}\;i=0\\ k_{i-1}\pm \text{rand}\times \text{DOI} & \text{if}\;i<360,i\in N, \end{array} \end{array}$$

where

(6) $$\begin{array}{c} \left|k_{0}-k_{359}\right|\leq \text{DOI}. \end{array}$$

The DOI describe the maximum variance of path loss at every direction. Equation (4) requires angle information between communicating nodes to provide path loss in all directions and we have to generate 360 *k*_*i*_ values for 360 different directions; therefore, its implementation is very complex. Hence, in [14], Xiao et al. proposed a simpler model which does not needed the information about angle between two sensor nodes; thus, DOI-adjusted path loss can be expressed as

(7) $$\begin{array}{c} \text{PL}{\left(d\right)}_{\text{DOI}}=\text{PL}\left(d\right)\times \left(1\pm \text{rand}\times \text{DOI}\right). \end{array}$$

The received signal strength *P*_*r*_(*d*) at distance *d* using DOI-adjusted path loss is

(8) $$\begin{array}{c} P_{r}\left(d\right)=P_{s}-\text{PL}{\left(d\right)}_{\text{DOI}}+P_{f}, \end{array}$$

where *P*_*s*_ the transmitted signal is power of the sender node and *P*_*f*_ is the fading exponent. Therefore, from (8), the probability of received signal when its power level exceeds certain threshold is

(9) $$\begin{array}{c} \text{Pr}\left(P_{r}\left(d\right)\geq \gamma \right)=\frac{1}{2}\mathrm{erfc}\left(\frac{\gamma -\bar{P_{r}\left(d\right)}}{\sigma \sqrt{2}}\right), \end{array}$$

where *γ* is the threshold of the received signal power. Using this probability described in (9), the sensor node calculates its communicating range, which is different for every node and depends upon shadowing effects.

## 4. *K*-Sensing Coverage

Coverage means that every point in the target region is observed by at least one sensor. This is called 1-coverage. To improve accuracy and to cope up with sensor failure, some applications, such as forest fire detection, intruder detection, and military surveillance, require *K*-coverage (*K* > 1), which means that every point in the region is monitored by at least *k* distinct sensor nodes. For obstacle free environments, the sensing radius of a sensor node is assumed to be constant in all directions, but, in reality, the sensing radius of sensor node is not uniform in all directions due to reflection, refraction and scattering caused by obstacles present in the environment. If the received signal strength is greater than a specific threshold value, then only the sensor node detects the sensing signal this is known as sensing sensitivity. If the average sensing radius of a node is $\bar{r}$, the sensing sensitivity *P*_senst_ of a sensor node is expressed as [9]

(10) $$\begin{array}{c} P_{\text{senst}}=P_{s}-\bar{L}\left(d_{0}\right)-10\eta \log_{10}\left(\frac{\bar{r}}{d_{0}}\right), \end{array}$$

where *P*_*s*_ is sending power, *d*_0_ is reference distance, and *η* is path loss component.

### 4.1. *K*-Sensing Coverage without Shadowing Impact

Let *r*_max_ and *A* be the effective sensing radius when *σ* = 0 of a sensor node and area of interest, respectively. Target in area *A* will be detected by any randomly deployed sensor if it is within sensing radius (*r*_max_) from the event. The probability that the target will be detected by an arbitrary sensor is

(11) $$\begin{array}{c} P=\frac{\pi r_{\max}^{2}}{A}. \end{array}$$

The probability of the target not being sensed by a randomly deployed sensor is

(12) $$\begin{array}{c} P_{\text{undet}}=\left(1-P\right). \end{array}$$

Let *N* sensors be deployed randomly in the area of interest. The probability of the target not being sensed by any one of the sensor node is

(13) $$\begin{array}{c} P_{\text{undet}}={\left(1-P\right)}^{N},\\ P_{\text{undet}}={\left(1-\frac{\pi r_{\max}^{2}}{A}\right)}^{N}. \end{array}$$

The probability that the target will be detected by at least one of the *N* nodes is

(14) $$\begin{array}{c} P_{\det}=\left(1-P_{\text{undet}}\right),\\ P_{\det}=\left(1-{\left(1-\frac{\pi r_{\max}^{2}}{A}\right)}^{N}\right), \end{array}$$

where *r*_max_ is the effective sensing range of sensor node which can be calculated as [15]

(15) $$\begin{array}{c} r_{\max}=10^{\psi }e^{\xi }. \end{array}$$

With

(16) $$\begin{array}{c} \psi =\frac{\lambda }{10}\times \eta ,\;\;\xi ={\left(\ln\left(10\right)\times \frac{\sigma }{10}\times \eta \right)}^{2}. \end{array}$$

### 4.2. *K*-Sensing Coverage with Shadowing Impact

**Figure 2:**
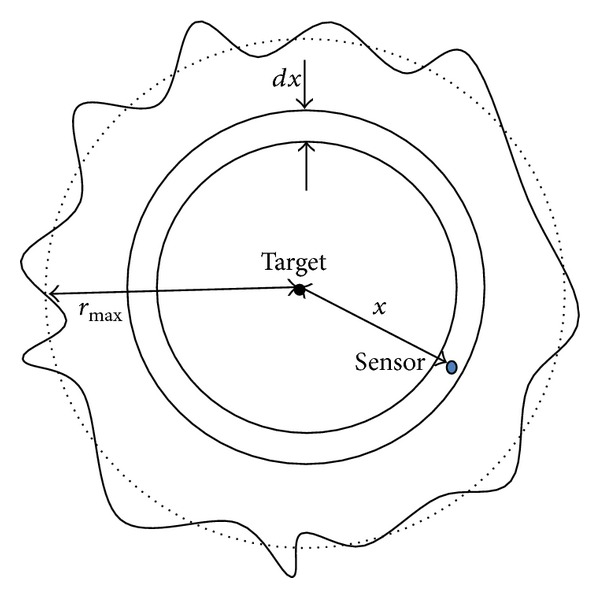
Impact of shadowed environment on sensing.

In a shadowing environment, sensing behavior of a sensor depends on the signal propagation and, therefore, sensing radius of a sensor node is not isotropic in all directions. Let us assume that a sensor node is deployed at a distance *x* from the target location as shown in Figure 2; therefore, the received power (in decibel units) can be expressed as

(17) $$\begin{array}{c} P_{r}\left(x\right)=P_{s}-P\bar{L}\left(d_{0}\right)-10n\log\left(\frac{x}{d_{0}}\right)+X_{\sigma }. \end{array}$$

A target is sensed by the sensor node when the received signal power is greater than some threshold value *γ*. The probability that the received power is greater than some threshold value can be expressed as

(18) $$\begin{array}{c} P_{\text{sens}}\left[P_{r}\left(x\right)>\gamma \right]=Q\left(\frac{\gamma -\bar{P_{r}\left(x\right)}}{\sigma }\right). \end{array}$$

Here, *Q* function is used to calculate the probability that the received signal will exceed some threshold value. Where *Q* function is expressed as

(19) $$\begin{array}{c} Q\left(z\right)=\frac{1}{\sqrt{2\pi }}\overset{\infty }{\underset{Z}{\int }}\exp\left(-\frac{x^{2}}{2}\right)dx. \end{array}$$

As sensor nodes are randomly deployed at a distance *x* over an area *A*. The probability that, a sensor node is randomly deployed, at a location with distance *x* to the event of interest, is 2*πx* *dx*/*A*, where *dx* is very small variation in distance. The probability that the event of interest is sensed by the sensor node is

(20) $$\begin{array}{c} P_{\det}=\overset{r_{\max}}{\underset{0}{\int }}P_{\text{sens}}\times \frac{2\pi x}{A}dx. \end{array}$$

Now, probability that a target is sensed by *P* sensors out of *N* sensors in sensing field of area *A* is

(21) $$\begin{array}{c} P_{K\mathrm{cov}}\left(P\right)=\left(\begin{array}{c} N\\ P \end{array}\right){\left(P_{\det}\right)}^{P}{\left(1-P_{\det}\right)}^{N-P}. \end{array}$$

Substituting the value of *P*_det_ from (20),

(22) PKcov(P)=(NP)(∫0rmaxQ(γ−Pr(x)¯σ)2πxAdx)P ×(1−(∫0rmaxQ(γ−Pr(x)¯σ)2πxAdx))N−P.

Probability that at least *K* sensors can cover the target location is given by

(23) PKcov(K) =1−∑P=0K−1(NP)×(∫0rmaxQ(γ−Pr(x)¯σ)2πxAdx)P×(1−(∫0rmaxQ(γ−Pr(x)¯σ)2πxAdx))N−P

From (23), we can find the probability of *K*-coverage; that is, each and every point in the field is detected by at least *K* sensor nodes so that the network is made fault tolerable. It can also be easily seen from the equation that as the shadowing parameter increases, the probability of coverage decreases.

## 5. *K*-Connectivity

In Wireless Sensor Networks, each sensor node sends its sensing data to the sink node by single or multi hop communication. So, one of the most significant property of WSN is the connectivity property that we will study in this subsection. The connectivity of a sensor network is affected by time, as time goes on, the energy of the sensor node depletes and sensor node fails and the network may be disconnected. *K*-connectivity is an important QoS measure of network for fault tolerant system and thus enhances the communication reliability. *K*-connectivity refers to the property of a randomly selected sensor node that has at least *K* neighbors. That is, in *K* connectivity, if (*K* − 1) node fails, then connectivity still holds. Since wireless channel is affected from various environment impairments, therefore *K*-connectivity performance is evaluated under log-normal shadowing model.

### 5.1. The Geometric Random Graph Model

A random graph is denoted by *Gp*(*n*), where *n* is the number of nodes and *p* is the probability of having link between any two nodes. The degree of a node in a network is defined as the number of connections or links the node has to other nodes. In a random graph, each of *n* nodes is connected with probability *p* and not connected with probability (1 − *p*) that has binomial distribution degrees *k*

(24) $$\begin{array}{c} P\left(k\right)=\left(\begin{array}{c} n-1\\ k \end{array}\right)P^{k}{\left(1-p\right)}^{n-1-k}. \end{array}$$

For large value of *n* and small value of *k*, we can use the following approximations:

(25) (n−1k)=(n−1)!k!(n−1−k)!(n−1)!k!(n−1−k)! =(n−1)(n−1−1)(n−1−2)⋯(n−1−k)!k!(n−1−k)!.

Therefore,

(26) $$\begin{array}{c} \left(\begin{array}{c} n-1\\ k \end{array}\right)=\frac{{\left(n-1\right)}^{k}}{k!}. \end{array}$$

The Poisson approximation for large value of *n* is given by

(27) P(k)=(n−1k)Pk(1−p)n−1−k=(n−1)kk!pke−m=e−mmkk!,

where *m* is the mean node degree *m* = *p*(*n* − 1).

### 5.2. *K*-Connectivity in Presence of Shadowing

Let us consider a wireless sensor network, where *N* sensor nodes are randomly distributed in the area *A* according to a homogeneous Poisson process with average intensity *λ*. The number of nodes per unit area is given by *λ* = *N*/*A*. The area of a sensor node with radius *r*_max_ is *A*_*s*_ = *πr*_max_^2^. The probability that a randomly selected sensor node with area *A*_*s*_ has *K* neighbors is given by the following equation:

(28) $$\begin{array}{c} \begin{array}{c} P\left(k\right)=\frac{{\left(\lambda A_{s}\right)}^{k}}{k!}\cdot e^{-\lambda A_{s}},\\ P\left(k\right)=\frac{{\left(\lambda \pi r_{\max}^{2}\right)}^{k}}{k!}\cdot e^{-\lambda \pi r_{\max}^{2}}, \end{array}\;\text{where}\;\left(k=1,2,3,\ldots \right). \end{array}$$

Now, the probability that a randomly selected sensor node has no neighbor, that is, the sensor node is isolated and the network is not connected

(29) $$\begin{array}{c} P\left(k=0\right)=e^{-\lambda \pi r_{\max}^{2}}. \end{array}$$

If a sensor node is isolated it cannot exchange information between other nodes and, thus, it is useless for the entire network. Penrose in [16] proved that there is a relationship between *K*-connectivity and the minimum node degree; that is, *p*(*K*-connectivity) ≈ *p*(*d*_min_ ≥ *K*). Therefore the expression for minimum node degree can be directly applied to *K*-Connectivity. Due to shadowing effect, the range of the received signal is not symmetric in all directions. Receivers located at the same distance from the sender may have different values for received signal. The probability that each node with effective transmission range *r*_max_ is *K*-connected can be given by the following expression:

(30) p(dmin≥K)=(1−e−λπrmax2×(1+λπrmax2+(λπrmax2)22+⋯+(λπrmax2)k−1k−1!))p(dmin≥k)=(1−e−λπrmax2∑i=0k−1λπrmax2i!)N,

where *N* is no. of nodes deployed, *λ* is node density, and *r*_max_ is effective communication range.

## 6. Effect of Node failure on *K*-Sensing Coverage 

Sensor nodes were scattered in remote and hostile environment, where there is no human involvement to maintain sensor nodes. Some nodes may be failed due to noise, hardware malfunction, software problem, and battery energy depletion and these nodes cannot be repaired. Due to failure of nodes, coverage ratio may be decreased and network may be partitioned which results in network failure.

Let *P*_nf_ be the probability of node failure and let *P*_det_ be the probability of detection of an event. The probability that there is no node failure is (1 − *P*_nf_). Now, suppose that *N* nodes are deployed to detect the target; the probability that out of *N* nodes no node detects the event in presence of node failure is given by

(31) $$\begin{array}{c} P_{0}={\left(1-P_{\det}\left(1-P_{\text{nf}}\right)\right)}^{N}. \end{array}$$

The probability that out of *N* nodes *k* nodes detect the event in presence of node failure probability *P*_nf_ is given as follows:

(32) $$\begin{array}{c} P_{k}=\left(\begin{array}{c} N\\ k \end{array}\right){\left(P_{\det}\left(1-P_{\text{nf}}\right)\right)}^{k}{\left(1-P_{\det}\left(1-P_{\text{nf}}\right)\right)}^{N-k}. \end{array}$$

Substituting the value of *P*_det_ from (15),

(33) Pk=(Nk)(∫0rmaxPsens×2πxAdx(1−Pnf))k ×(1−∫0rmaxPsens×2πxAdx(1−Pnf))N−k.

From this equation, we can find out the probability of *k* coverage in presence of node failure and shadowing.

## 7. Simulation Results and Performance Analysis 

In this section, we present a performance analysis of network for *K*-Coverage and *K*-Connectivity. All nodes use the log-normal shadowing model for sensing and communication. Simulations are performed using MATLAB to investigate the *K*-Coverage and *K*-Connectivity results. Different values of simulation parameters used to conduct experiments are tabulated in Table 2. To show the effect of node density and shadowing parameters, we have conducted the following experiments:

- effect of node density and standard deviation on sensing coverage,
- effect of node failure on sensing coverage,
- effect of node density on connectivity,
- effect of shadowing parameter on connectivity,
- effect of communication range on connectivity,
- effect of node distance on connectivity.

Below, we conducted all these experiments one by one.

**Figure 3:**
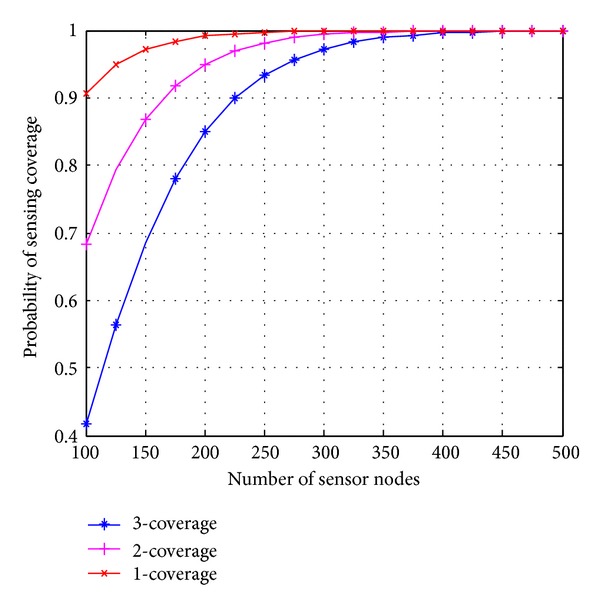
Probability of coverage versus sensor nodes, standard deviation *σ* = 2.

**Figure 4:**
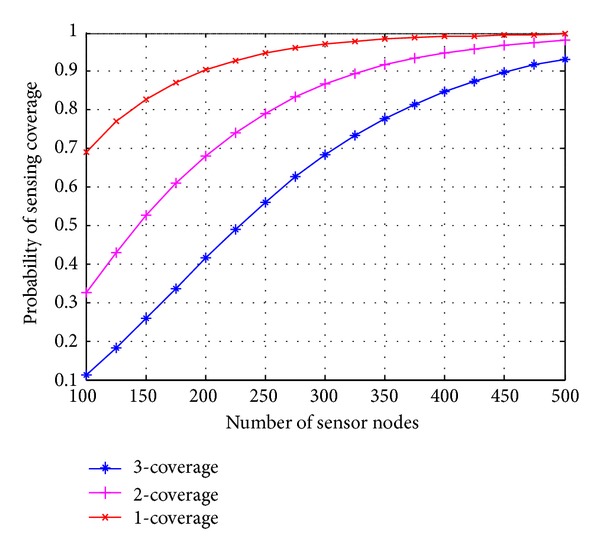
Probability of coverage versus sensor nodes, standard deviation *σ* = 4.

*(i) Effect of Node Density and Standard Deviation on Sensing Coverage.* Figures 3 and 4 show the result of the probability of coverage versus the number of sensor nodes for different shadowing parameters (*σ* = 2, 4). When the standard deviation of the shadowing environment is increased from 2 to 4, the average sensing radius of sensor node also decreases, and this requires more number of sensor nodes to achieve desired coverage. As shown in Figure 3, in order to provide 3-coverage with probability >90% and shadowing parameter *σ* = 2, approximately 225 sensor nodes are required in the area *A* of 100 × 100. When the shadowing parameter increases from 2 to 4, approximately 450 nodes are required to achieve 3-Coverage with the same probability. Slope of the curve in Figure 3 is bigger as compared to Figure 4. This concludes that when the standard deviation increases, probability of coverage is not increased in the ratio of node density.

**Figure 5:**
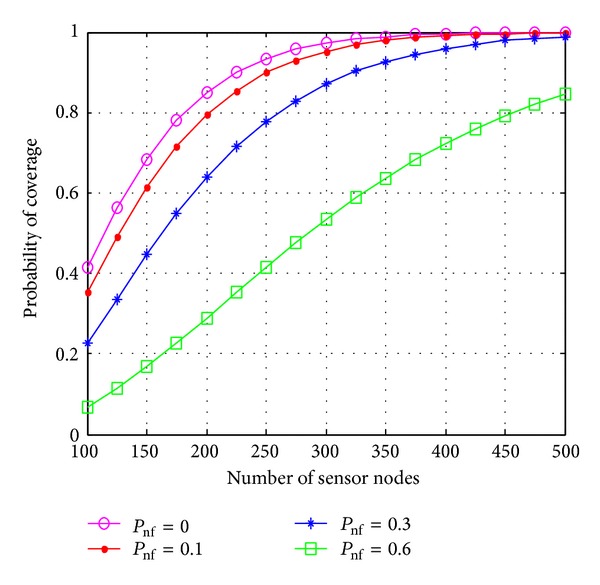
Probability of coverage versus number of sensor nodes for different failure probability.

*(ii) Effect of Node Failure on Sensing Coverage.* Figure 5 shows the probability of 3-coverage with different number of sensor nodes for four different failure probabilities. Here, we consider standard deviation parameter *σ* = 2 and path loss exponent *η* = 3. it is clear from the figure that when the number of sensor node increases, the probability of coverage also increases even in presence of node failure. When the probability of node failure is high, the slope of the curve is less. This indicates that adding extra nodes does not help much to increase the network coverage as compared to that when the failure probability of node is less.

**Figure 6:**
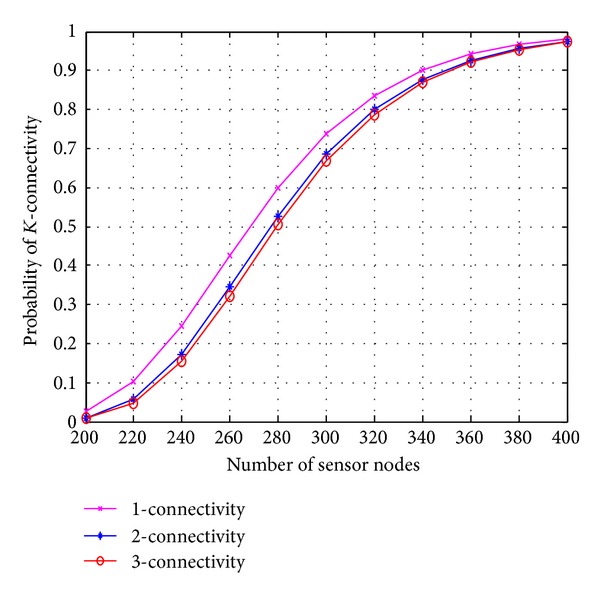
Probabaility of *K*-connectivity versus number of sensor.

*(iii) Effect of Node Density on Connectivity.* Figure 6 shows the result for *K*-connectivity with *N* nodes. When we deploy 200 nodes in a square region of 100 × 100, the network is almost disconnected. For surely connected network with probability ≥90%, we require approximately 340 sensor nodes. If we deploy 360 nodes in the same area, we will get 3-connected network with probability >90%. Therefore, as the density of the sensor node increases, the graph shows transition from low connectivity to high connectivity.

**Figure 7:**
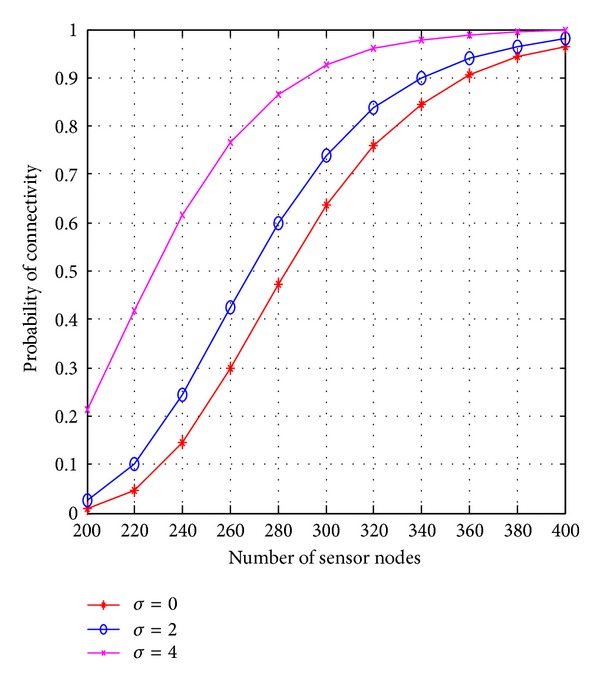
Probability of network connectivity versus sensor nodes.

*(iv) Effect of Shadowing Parameter on Connectivity.* Figure 7 shows the relationship between network connectivity, number of sensor nodes, and standard deviation constant. For surely connected network with probability >90%, we require approximately 360 nodes in the square region of 100 × 100 when there is no fading. If we increase standard deviation constant *σ*, less number of nodes are sufficient to make the network connected with same probability. When the standard deviation constant increases then irregularity in the communication range also increases, that is, communication link is more asymmetric. A less number of shorter links are removed and large numbers of longer links are added because the number of nodes increases linearly with distance. Therefore a node which is closer to the communication range may be isolated but in turn some more nodes that are farther away may get connected. As shown in Figure 4, when the Standard deviation *σ* = 2, we require 340 nodes, and when the *σ* = 4, approximately 296 nodes are sufficient to get network connected with same probability. Hence, the probability of *K*-connectivity increases with the presence of log-normal shadowing.

**Figure 8:**
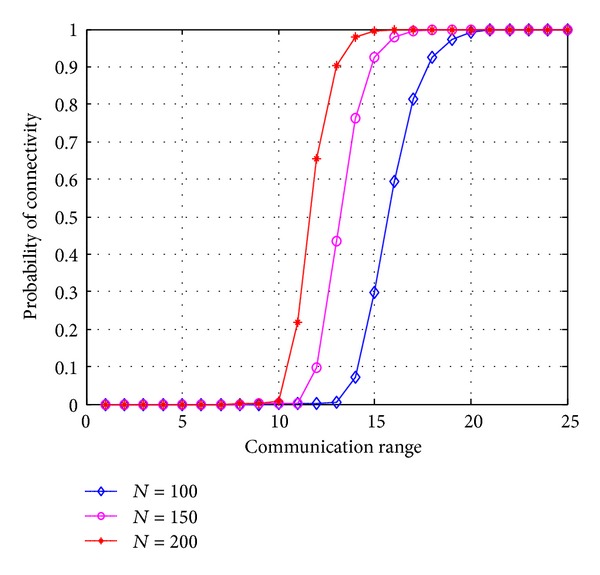
Probability of connectivity versus communication range.

*(v) Effect of Communication Range on Connectivity.* Figure 8 shows probability of connectivity as a function of communication range. Here, we can see the effect of communication range on the network connectivity. The connectivity of the network increases when the communication range of the nodes increases. When the communication range of the sensor nodes is less than 10 m, the network is almost disconnected. As the communication range of the nodes increases from 10 m to 13 m, the connectivity also increases and reaches to approximately 90% for the communication range 13 m and *N* = 200. For less number of nodes *N* = 100 and same high probability of connectivity, the communication range is 18 m approximately as shown in Figure 8.

**Figure 9:**
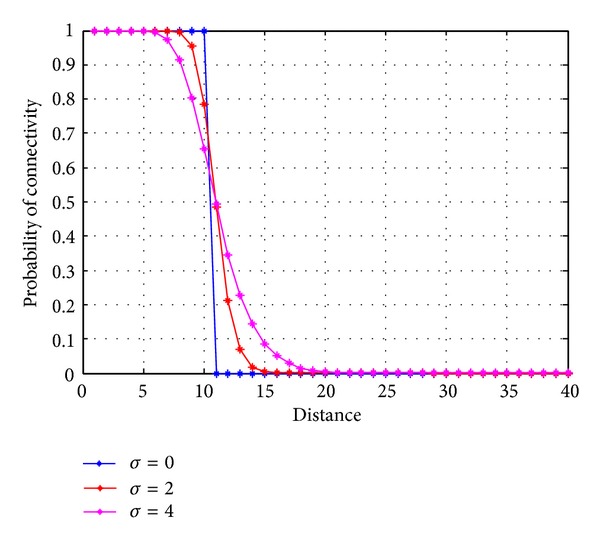
Probability of connectivity versus distance.

*(vi) Effect of Nodes Distance on Connectivity.* Figure 9 shows the probability of Connectivity with the distance between two sensor nodes. It can be observed from Figure 6 that, when the standard deviation *σ* = 0 and distance between two nodes is 11 m then probability of connectivity is almost zero, but when the shadowing increases from 0 to 2 and 4, the probability of the link between two nodes is approximately 60% and 70%, respectively, for the same distance.

## 8. Conclusions

One important QoS parameter in the design of WSN is that the network should be fault tolerance. In this paper *K*-coverage and *K*-connectivity are evaluated under log-normal shadowing model. Further we have studied the random shadowing effects which are incorporated into path loss and better describe received signal strength with distance in real environments. We have also shown the effect of node density and node failure probability on coverage. It has been observed that the probability of network coverage and connectivity depends on the node density and standard deviation. A higher value of standard deviation results in the decrease of average sensing radius of sensor node. Therefore, the probability of network coverage degrades. On the other hand high fading variance adds more links as compared to removed links thus improves connectivity. It can be concluded that log-normal path loss model and node density have a significant impact on coverage and connectivity.

## Figures and Tables

**Table 1:** Parameters definitions.

Parameter	Definition
*N*	Number of sensor nodes
*λ*	Node density
*P*_*s*_	Transmission power of a sensor node
*η*	Path loss exponent
*d*_*o*_	Close in reference distance
*σ*	Standard deviation
*P*_senst_	Sensing sensitivity of sensor node
*γ*	Threshold of received signal
*A*	Area of interest
*r*_max_	Effective sensing range
*P*_nf_	Probability of node failure
*P*_det_	Probability of detection of event

**Table 2:** Simulation parameter.

Parameter	Value
Number of sensor nodes (*N*)	500
Area (*A*)	10000 m^2^
Path loss exponent (*η*)	3
Transmission power of a sensor node (*P*_*s*_)	20 W
Threshold of received signal (*γ*)	−58 dB
Effective sensing range (*r*_max_)	10 m
